# Mitochondrial Reactive Oxygen Species Are Essential for the Development of Psoriatic Inflammation

**DOI:** 10.3389/fimmu.2021.714897

**Published:** 2021-08-06

**Authors:** Soichi Mizuguchi, Kazuhito Gotoh, Yuya Nakashima, Daiki Setoyama, Yurie Takata, Shouichi Ohga, Dongchon Kang

**Affiliations:** ^1^Department of Clinical Chemistry and Laboratory Medicine, Graduate School of Medical Sciences, Kyushu University, Fukuoka, Japan; ^2^Department of Pediatrics, Graduate School of Medical Sciences, Kyushu University, Fukuoka, Japan

**Keywords:** C1qbp/p32, dendritic cells, mitochondrial ROS, IL-1β, psoriasis

## Abstract

Psoriasis is a common immune-mediated, chronic, inflammatory skin disease that affects approximately 2–3% of the population worldwide. Although there is increasing evidence regarding the essential roles of the interleukin (IL)-23/IL-17 axis and dendritic cell (DC)-T cell crosstalk in the development of skin inflammation, the contributions of mitochondrial function to psoriasis are unclear. In a mouse model of imiquimod (IMQ)-induced psoriasiform skin inflammation, we found that hematopoietic cell-specific genetic deletion of p32/C1qbp, a regulator of mitochondrial protein synthesis and metabolism, protects mice from IMQ-induced psoriatic inflammation. Additionally, we demonstrate that p32/C1qbp is an important regulator of IMQ-induced DC activation, both *in vivo* and *in vitro*. We also found that p32/C1qbp-deficient DCs exhibited impaired production of IL-1β, IL-23, and mitochondrial reactive oxygen species (mtROS) after IMQ stimulation. Because the inhibition of mtROS suppressed IMQ-induced DC activation and psoriatic inflammation, we presume that p32/C1qbp and mtROS can serve as therapeutic targets in psoriasis.

## Introduction

Psoriasis is a skin disease that affects approximately 2–3% of the world population, including 10% of the population in some Northern European countries (1–4). Psoriasis is a common, chronic, relapsing inflammatory dermatitis, which is characterized by epidermal hyperplasia, erythematous plaque formation, and inflammatory cell infiltration in the dermis and epidermis (5, 6). Although the complex pathogenesis of psoriasis remains poorly understood, there is increasing evidence that immune function plays pivotal roles in disease development (5, 7, 8). In the past decade, clinical studies have shown that cytokines released by interleukin-17 (IL-17)-producing T cells (Th17) and dendritic cells (DCs) (e.g., IL-17, tumor necrosis factor-α, IL-12/23, and IL-22) are critical for the development of psoriasis (9, 10). In addition, multiple biologic agents (including monoclonal antibodies targeting IL-17, tumor necrosis factor-α, and IL-12/23) are available for the treatment of moderate to severe psoriasis (11, 12). Although biologic agents have robust effects on psoriasis treatment, various side effects associated with the administration have become an important problem in recent years (13). Thus, there is a need for novel psoriasis-targeting therapeutic agents.

Psoriasis is a disorder of both innate and adaptive immune systems, in which dendritic cells (DCs), keratinocytes, and T cells have central roles. DCs are key sentinels of the immune system, which bridge the gap between innate and adaptive immunity. Thus far, several studies have indicated that DCs contribute to the initiation of psoriasis through antigen-presenting molecules (e.g., major histocompatibility complex [MHC], MHCII, CD86, and CD40), cytokines (e.g., interferon [IFN], IL-1β, IL-23, and IL-6), and chemokine receptors (6, 11, 14). The proportions of myeloid DCs (mDCs) are increased in psoriasis lesions. In addition, mDCs from psoriasis lesions reportedly activated T cells to produce both IL-17 and IFN-γ. These mDCs also regulated the IL-23/IL-17 axis (15, 16). Furthermore, DCs activated by Toll-like receptor (TLR) 7-9 stimulation can trigger the onset of autoimmune diseases, including psoriasis and systemic lupus erythematosus (17–20). Although previous studies showed that the TLR7/Th17 axis contributes to psoriatic inflammation (21–23), the detailed molecular mechanism is unknown.

Imiquimod (IMQ) is a TLR 7/8 agonist that has been widely used for the topical treatment of human papilloma virus infection (17, 24, 25). In humans, IMQ can induce *de novo* psoriasis lesions and exacerbate preexisting psoriasis lesions (17, 26). In addition, daily topical application of IMQ cream on the skin and ears of mice leads to specific dermatitis with many features similar to human psoriasis manifestations (27, 28). Because the disease in this mouse model is heavily dependent on the IL-23/IL-17 axis, similar to human psoriasis (27, 28), these mice have been widely used for psoriasis-related pathological analysis and drug development. Recent studies have shown that IMQ directly or indirectly affects the mitochondrial electron transport chain (ETC), reactive oxygen species (mtROS), metabolism *in vitro* (29, 30).

The complement component 1q subcomponent binding protein (C1QBP/p32/HABP1) was originally identified as a protein associated with the essential splicing factor ASF/SF2 (31). Our group showed that p32/C1qbp is processed by proteolytic cleavage of N-terminal amino acids containing the mitochondrial signal sequence; moreover, it is essential for mouse embryonic development dependent on mitochondrial translation (32, 33). In macrophages and DCs, we demonstrated that p32/C1qbp is an important innate immunity factor (34, 35). Recent reports have shown that p32/C1qbp mutations impair mitochondrial respiratory chains and cause cardiomyopathy in humans and mice (36, 37). Despite the importance of mitochondrial function, the exact mechanisms of p32/C1qbp in psoriasis remain unclear.

Various genetic, environmental, and immunological factors reportedly contribute to psoriasis development (8, 38, 39). However, the roles of mitochondria in psoriasis pathogenesis are poorly understood (40). To explore the roles of mitochondria in psoriasis pathogenesis, we employed an IMQ-induced psoriasiform skin disease model. We hypothesized that p32/C1qbp is involved in immune cell-mediated exacerbation of psoriasis (e.g., *via* DCs and T cells). Therefore, we developed hematopoietic cell-specific p32/C1qbp-deficient mice. In the present study, we show that p32/C1qbp is involved in psoriasis pathogenesis through DC activation.

## Methods

### Animals

C57BL/6 mice were purchased from Japan Clea. Vav1–iCre mice (cat. no. 008610) were obtained from Jackson Laboratory. p32^flox/flox^ mice have been described previously (33). Age- and sex-matched p32^flox/flox^ Vav1–iCre^+^ (p32 cKO) and control littermate p32^flox/flox^ Vav1–Cre^–^ (WT) mice were used in this study (41). All mice were maintained on the C57BL/6 background and housed under specific pathogen-free conditions in the animal facility at Kyushu University. The animal research protocols were approved by the Committee of Ethics on Animal Experiments, Faculty of Medical Sciences, Kyushu University.

### Cell Cultures

Bone marrow DCs (BMDCs) were prepared as described elsewhere (42). To generate BMDCs, bone marrow cells were cultured for 6–8 days with GM-CSF (10 ng/mL; PeproTech) in complete medium, comprising RPMI 1640 medium (Sigma-Aldrich) supplemented with 10% FBS, penicillin (Thermo Fisher Scientific), streptomycin (Thermo Fisher Scientific), L-glutamine (Thermo Fisher Scientific), non-essential amino acids (Thermo Fisher Scientific), sodium pyruvate (Thermo Fisher Scientific), and 2-mercaptoethanol (Wako Pure Chemical Industries).

### *In Vivo* Treatments

IMQ-induced psoriasis-like inflammation was elicited and skin severity was assessed as previously described (27). For the IMQ-induced psoriasis model, a daily dose of 33–50 mg of IMQ-containing cream, Aldara (Beselna Cream 5%, Mochida Pharmaceuticals), was applied onto each mouse ear for 4–9 consecutive days. Skin severity was evaluated using the PASI scoring system based on the extent of skin thickness, redness, and scaling. Mitoquinone (MitoQ) is a mitochondria-targeted antioxidant that reduces mtROS (43). C57BL/6 mice were injected intraperitoneally with MitoQ (10 mg/kg, Focus Biomolecules) or mock treatment on days 1, 3, 6, and 8.

### Skin Histology

Skin sections were stained with H&E. Epidermal thickness was determined by measuring the mean interfollicular distance under the microscope. For IHC staining, skin cryosections were fixed, blocked, and stained with purified rabbit-anti-mouse Ki-67 Ab (1:200 dilution), followed by anti-rabbit IgG secondary antibody (1:200 dilution). Slides were developed with AEC substrate solution (Vector Laboratories) and then counterstained with hematoxylin. Images were acquired at 200× magnification using a Keyence BZ-X800 microscope.

### Quantitative Real-Time PCR Analyses

After stimulation or treatment with IMQ (5 μg/mL), BMDCs and mouse ears were collected and washed in PBS, then resuspended in RLT buffer. Total RNA was extracted from BMDCs and mouse ears using an RNeasy Mini Kit (Qiagen), in accordance with the manufacturer’s instructions. RNA concentration (ng/μL) and sample purity (260/280 ratio) were measured using the NanoDrop 1000 Spectrophotometer (Thermo Fisher Scientific). Reverse transcription of approximately 500 ng of total RNA was performed with PrimeScript RT reagent Kit (Takara, Japan), in accordance with the manufacturer’s protocol. Gene expression was measured by SYBR Green-based qPCR. Relative quantification was performed using the comparative cycle threshold (Ct) method, relative to 18S ribosomal RNA. PCR primers are listed in **Supplementary Table S1**.

### FACS

Mouse ears were collected and split into dorsal and ventral halves. They were then floated for 40 min at 37°C on the surface of 0.5% trypsin (w/v), which facilitated separation of the dermis from the epidermis. The dermis was cut into small pieces and placed into digestion solution containing collagenase type IV (1.5 mg/mL). Digestion was performed for 90 min at 37°C with mixing. After the completion of digestion, the solution was mixed thoroughly and filtered through a 70-μm nylon filter to obtain a single-cell suspension. For analysis of surface markers, cells were stained in PBS containing 2% (v/v) fetal bovine serum with cell surface antibodies. For intracellular staining with antibodies, cells were fixed with 4% (wt/vol) paraformaldehyde (Wako Pure Chemical Industries) and permeabilized with saponin (0.5% saponin and 0.5% BSA in PBS) for 15 min at room temperature.

BMDCs were stimulated with IMQ (5 and 25 µg/mL) with or without MitoQ (5 and 10 µM). Reagents were added to cells, simultaneously with imiquimod. Antibodies are listed in **Supplementary Table S2**. Flow cytometry analysis was performed with a FACSVerse using FACSuite software (BD Biosciences).

### *In Vitro* Dermal γδ T Cell Stimulation

Whole skin cell suspensions from the ears of WT or p32cKO mice were cultured in cRPMI in the presence of mouse IL-23 (15 ng/mL, BioLegend), IL-1β (30 ng/mL, PeproTech), or IL-23 and IL-1β for 72 h. Brefeldin A (5 µg/mL, BioLegend) was added 2–3 h before intracellular staining and flow cytometry.

Whole skin cell suspensions from the ears of WT or p32cKO mice treated with IMQ for 4 consecutive days were re-stimulated with phorbol myristate acetate (PMA, 1 µg/mL, Wako Pure Chemical Industries) and ionomycin (0.1 µg/mL, Wako Pure Chemical Industries) in the presence of brefeldin A (5 µg/mL, BioLegend) for 2–3 h. They were then stained by the intracellular staining procedure as described to determine IL-17A expression (35).

### Mitochondrial ROS Production, Mass, and Membrane Potential

BMDCs were incubated with 1 µM MitoSOX Red mitochondrial superoxide indicator (Thermo Fisher Scientific) at 37°C for 15 min in HBSS, washed two times with HBSS, and then analyzed with flow cytometry.

BMDCs were incubated in HBSS with 5 or 25 µM IMQ for 10–15 min at 37°C. MitoSOX Red (1 µM, Invitrogen), MitoTracker Green FM (50 nM, Invitrogen), and TMRM (100 nM, Invitrogen) were respectively added at the beginning of incubation. BMDCs were washed two times with HBSS, then analyzed with flow cytometry.

### Metabolism Assays

BMDCs were analyzed using an XF-24 Extracellular Flux Analyzer (Seahorse Bioscience) (44). Briefly, BMDCs were seeded in XF-24 well culture plates (200,000 cells/well). At the specified time points, BMDCs were washed and analyzed in XF Running Buffer (unbuffered RPMI medium with 10 mM glucose, 10% fetal calf serum, and 2 mM L-glutamine), in accordance with the manufacturer’s instructions, to obtain real-time measurements of the OCR and ECAR. Analyses of the ECAR and/or OCR in response to 5 or 25 μg/mL IMQ were performed.

### Immunoblotting Analysis

For direct immunoblotting, BMDCs were washed in PBS, then lysed with cell lysis buffer (Cell Signaling Technology). The lysate was eluted with sample buffer and boiled for 5 min at 97°C. SDS-PAGE was performed with 8%–15% polyacrylamide gels using a constant current of 20 mA. The gels were transferred to Immobilon-P membranes (Merck Millipore) using a semidry blotting device for 15 min, with a constant current of 2.5 A. The membranes were blocked with Blocking One (Nacalai Tesque) and incubated with the appropriate primary antibody overnight at 4°C in Can Get Signal Solution 1 (Toyobo Co., Ltd.) (**Supplementary Table S2**). The membranes were subsequently incubated with the appropriate secondary antibody for 1 hour at RT in Can Get Signal Solution 2 (Toyobo Co., Ltd.), then washed again. Signals were visualized by chemiluminescence using a Clarity ECL Substrate (BIO-RAD) and an ImageQuant LAS4000 Mini image analyzer (GE Healthcare), in accordance with the manufacturer’s protocols.

### ELISA

For cytokine quantification of cell-free supernatants, ELISA kits for murine IL-1β and IL-23 from BioLegend were used as directed by the manufacturer. Data are expressed as the means ± SDs of triplicate wells.

### Quantification and Statistical Analysis

Statistical analyses were performed using GraphPad Prism. All experiments were performed with at least two independent biological replicates. The results are expressed as the means ± SDs or means ± SEMs of the independent experiments. P-values were calculated using two-tailed Student’s t-tests.

## Results

### Absence of p32/C1qbp in Hematopoietic Cells Protects Mice From IMQ-Induced Psoriatic Inflammation

**Figure 1:**
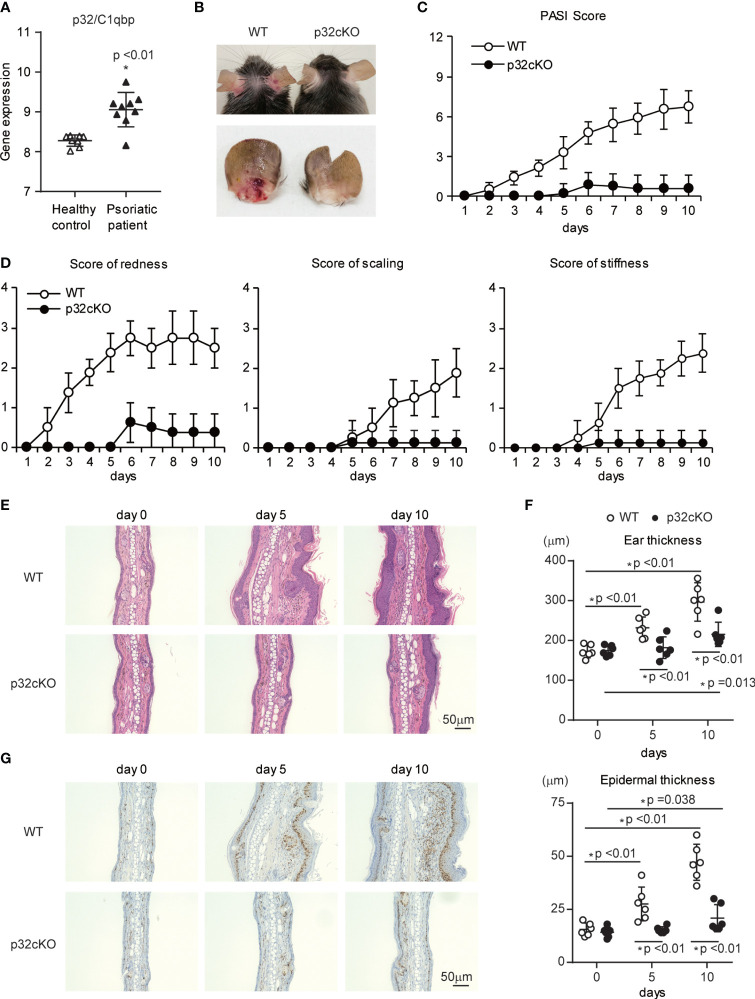
Effects of p32/C1qbp deficiency on psoriatic inflammation. **(A)** Gene expression patterns of p32/C1qbp in lesions from patients with psoriasis (n = 9) versus healthy individuals (n = 8) from Gene Expression Omnibus (http://www.ncbi.nlm.nih.gov/geo; accession number GSE75890). **(B–D)** WT and p32cKO mice were topically treated with IMQ for 9 consecutive days. Representative gross appearances of ears from WT and p32cKO mice that were treated daily with topical IMQ **(B)**. Cumulative Psoriasis Area Severity Index (PASI) scores **(C)** were calculated based on the individual scores for redness, scaling, and stiffness **(D)** daily until 9 days after IMQ application. Data are shown as means ± SDs (n = 8). **(E–G)** Microscope images of cross-sections of mouse ears stained with hematoxylin and eosin **(E)** and subjected to immunohistochemical analysis of Ki-67 **(G)**. Changes in ear (**F** upper) and epidermal (**F** lower) thickness over time. Scale bars, 50 µm. Data are shown as means ± SDs **(A, C, D**, **F)**. *p < 0.05 versus WT mice. Data are representative of at least three **(A–G)** independent experiments.

We found that the gene expression of p32/C1qbp was significantly increased in lesional skin from patients with psoriasis, compared with healthy controls (38) (**Figure 1A**). Thus, we investigated the relationship between p32/C1qbp and IMQ-induced psoriatic inflammation using a mouse model. The gene expression of p32/C1qbp was increased during IMQ-induced psoriatic inflammation, similar to human psoriasis (**Supplementary Figure 1A**). To evaluate the role of immunological p32/C1qbp in psoriatic inflammation, we generated a hematopoietic-specific p32/C1qbp conditional knockout (cKO) mouse strain by mating loxP-flanked p32/C1qbp gene mutation p32^flox/flox^ mice with Vav1–Cre transgenic mice. Repetitive topical application of IMQ to the ears of WT mice (Vav1–Cre^–^ p32^flox/flox^ mice) induced psoriatic inflammation with marked erythema, scaling, and stiffness (**Figures 1B–D**). Notably, p32cKO mice had reduced the severity of psoriatic symptoms, compared with WT mice (**Figures 1B–D**). Histological examination also revealed that IMQ-treated skin in WT mice was characterized by epidermal hyperplasia, with massive infiltration of inflammatory cells (**Figures 1E, F** and **Supplementary Figure 1B**). In contrast, p32cKO mice did not exhibit these histological changes following IMQ treatment (**Figures 1E, F**). The increased ear thickness following IMQ application in WT mice was abrogated in p32cKO mice (**Figure 1F**). Additionally, the proportion of epidermal keratinocytes expressing the proliferation marker Ki-67 was smaller in skin from IMQ-treated p32cKO mice, compared with skin from IMQ-treated WT mice (**Figure 1G**). These results demonstrate that p32/C1qbp in hematopoietic cells is essential for the development of IMQ-induced psoriatic dermatitis.

### p32/C1qbp Regulates IMQ-Induced Psoriatic Inflammation

**Figure 2:**
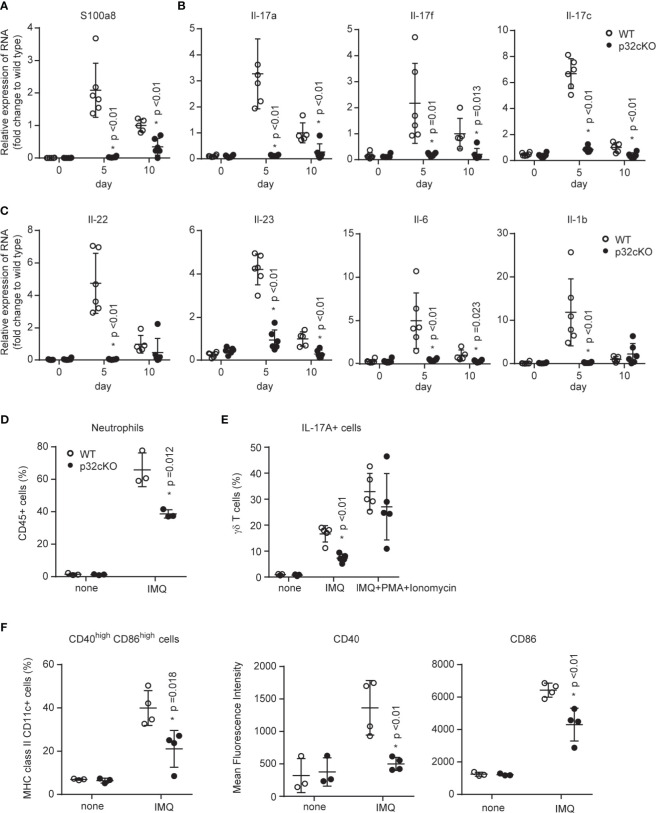
Roles of p32/C1qbp in psoriatic inflammation *in vivo*. **(A–C)** WT and p32cKO mice were treated with IMQ for 9 consecutive days. Real-time PCR analysis was performed to assess mRNA levels in IMQ-treated mouse ears on days 5 and 10 (n ≥ 3). The results were normalized to 18S expression and are shown as means ± SDs. **(D–F)** WT and p32cKO mice (n ≥ 3) were treated with IMQ for 4 consecutive days. Infiltration of neutrophils **(D)**, IL-17^+^ γδ T cells **(E)**, and CD40^+^ CD86^+^ DCs populations in the ears were analyzed by flow cytometry. IL-17^+^ γδ T cell numbers were analyzed before (**E**, left and middle lanes) and after (**E**, right lane) PMA/ionomycin (PI) stimulation *in vitro*. Expression levels of CD40 and CD86 were measured in DCs obtained from the ears of WT and p32 cKO mice. Results are expressed as means ± SDs. Data are shown as means ± SDs **(A–F)**. *p < 0.05 versus WT mice. Data are representative of at least three **(A–F)** independent experiments.

Because the IL-23/IL-17 axis is an important pathway in psoriasis pathogenesis, we examined whether the loss of p32/C1qbp in hematopoietic cells inhibit the IL-23/IL-17-mediated pathogenesis of psoriasis-like dermatitis. Quantitative RT-PCR analysis revealed significantly reduced expression levels of typical psoriasis lesion-associated antimicrobial peptides (S100a8) in ears from p32cKO mice (**Figure 2A**). In addition, mRNA expression levels of proinflammatory cytokines (e.g., IL-17, IL-22, IL-23, IL-6, and IL-1β were significantly reduced in skin from IMQ-treated p32cKO mice (**Figures 2B, C**). Therefore, we concluded that p32/C1qbp in immune cells is a key driver in the development of IMQ-induced psoriatic dermatitis.

Psoriasis is a disorder of both innate and adaptive immune systems involving DCs, T cells, and neutrophils have central roles. Indeed, we found that neutrophils predominantly infiltrated into the ears from IMQ-treated WT mice (**Figure 2D** and **Supplementary Figure 2A**). In contrast, neutrophil infiltration was significantly reduced in the skin from p32cKO mice. Because the loss of p32/C1qbp did not affect neutrophil migration (**Supplementary Figure 2B**), the reduced neutrophil infiltration associated with p32/C1qbp deficiency may be dependent on other cells (e.g., DCs and γδ T cells). We also found that the proportion of IL-17-producing γδ T cells was decreased in the ears from IMQ-treated p32cKO mice (**Figure 2E**, middle lane, **Supplementary Figure 2C**). Because the induction of IL-17A by phorbol myristate acetate (PMA) + ionomycin stimulation occurred normally in γδ (**Figure 2E**, right lane) and αβ T cells (**Supplementary Figure 2D**) from p32cKO mice *in vitro*, we presumed that p32/C1qbp acts independently of a protein kinase C and Ca^2+^ signaling pathway in T cells. DCs became activated and promoted inflammatory responses after IMQ application. Indeed, we found that DCs from the ears of WT mice were activated after IMQ stimulation (**Figure 2F**, **Supplementary Figure 2E**). In contrast, DCs from the ears of p32cKO mice demonstrated impaired activation of CD40 and CD86 following IMQ stimulation (**Figure 2F**). To investigate whether DCs are directly involved in the exacerbation of psoriasis, we subcutaneously administered IMQ-treated WT or p32-deficient DCs to wild-type mice. At 48 hours after transfer, redness was significantly exacerbated in ears with IMQ-treated WT BMDCs (**Supplementary Figure 2F**), compared with IMQ-treated p32 deficient DCs. Therefore, we concluded that p32/C1qbp regulates the exacerbation of psoriatic inflammation through DC activation, rather than T cell activation and neutrophil migration.

### p32/C1qbp Is a Key Regulator of IMQ-Induced DC Activation *In Vitro*

**Figure 3:**
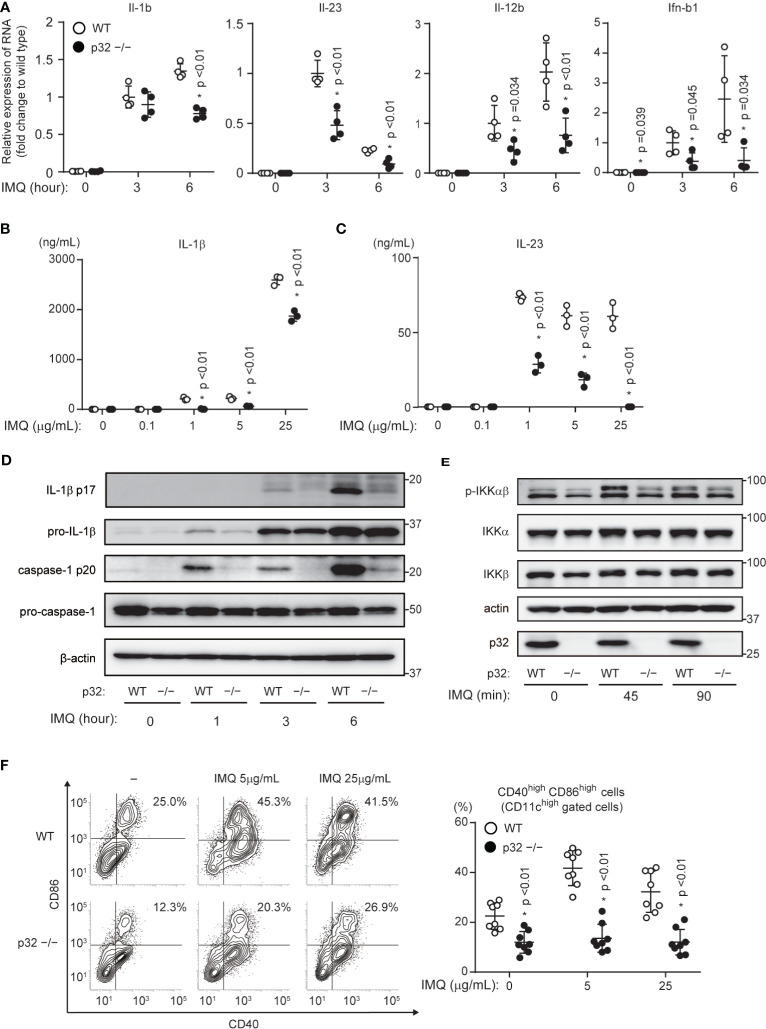
Roles of p32/C1qbp *in vitro*. **(A)** Real-time PCR analyses of Il-1b, Il-23, Il-12b, and Ifnb1 expression levels in WT and p32^−/−^ DCs stimulated with 5 μg/mL IMQ for the indicated intervals. Data are shown as means ± SEMs after normalization to expression of the gene encoding 18S ribosomal RNA (18S rRNA). **(B, C)** Levels of IL-1β **(B)** and IL-23 **(C)** in cell culture supernatants were compared between WT and p32^−/−^ BMDCs (2×10^5^ cells/well) after 24 h of stimulation with IMQ. Data are shown as means ± SDs of triplicate wells. **(D, E)** WT and p32^−/−^ DCs were stimulated with 5 μg/mL IMQ for the indicated intervals and analyzed for IL-1β p17, caspase-1 p20 **(D)**, and phosphorylation of IKK-αβ (E). β-Actin was used as an internal control. **(F)** Expression levels of cell surface markers of CD86 and CD40 in WT (upper) and p32^−/−^ (lower) BMDCs left untreated (−) or stimulated for 12 h with IMQ and analyzed using flow cytometry to quantify cell surface staining of CD86 and CD40. Numbers in top right corners indicate the percentages of CD86^+^ CD40^+^ cells. Data are representative of two **(A)** and three **(B–F)** independent experiments.

Because we thought that p32/C1qbp-dependent DC activation was associated with the exacerbation of psoriasis, we analyzed the relationship between p32/C1qbp and DC activation following IMQ stimulation *in vitro*. DCs express TLR7 and produce both proinflammatory cytokines and type I interferon (IFN) in an NF-κB or interferon regulatory factor (IRF)-dependent manner, following IMQ stimulation. Consistent with our *in vivo* data (**Figure 2C**), we found that IMQ-induced transcription and production of both IL-1β and IL-23 were impaired in BMDCs from p32cKO mice (p32^–/–^ DCs) (**Figures 3A–D**). In addition, the loss of p32/C1qbp suppressed the expression of Il-12b and Ifnb1 following IMQ stimulation (**Figure 3A**). Because phosphorylation levels of IKKα/β, IκB, and NF-κB were suppressed in p32^–/–^ DCs stimulated with IMQ (**Figure 3E** and **Supplementary Figure 3A**), we presumed that p32^–/–^ DCs exhibit reduced the production of proinflammatory cytokines. IL-1β and IL-23 induced IL-17 production from γδ T cells. Indeed, we found that γδ T cells from the ears of WT mice enhanced IL-17 production by stimulating IL-1β and IL-23 *in vitro* (**Supplementary Figure 3B** most right lane). Because IL-1β-induced IL-17 production by γδ T cell was unaffected by p32/C1qbp gene deficiency and IL-23 did not induce IL-17 production in γδ T cells (**Supplementary Figure 3B**, middle two lane), we speculated that psoriatic inflammation could be alleviated by reducing IL-1β production from p32^–/–^ DCs *in vivo*.

Although cytokines such as IL-17, IL-23, and IL-1β are mainly involved in the exacerbation of psoriasis, DC–T cell crosstalk through CD80/CD86 also affects psoriasis. We showed that p32/C1qbp was involved in DC maturation following IMQ administration *in vivo* (**Figure 2F**). The loss of p32/C1qbp in BMDCs led to suppression of DC maturation by IMQ stimulation *in vitro* (**Figure 3F** and **Supplementary Figures 3C, D**). These findings suggest that p32/C1qbp is associated with psoriasis pathology through DC activation.

### p32/C1qbp Supports Mitochondrial ROS Generation After IMQ Stimulation

**Figure 4:**
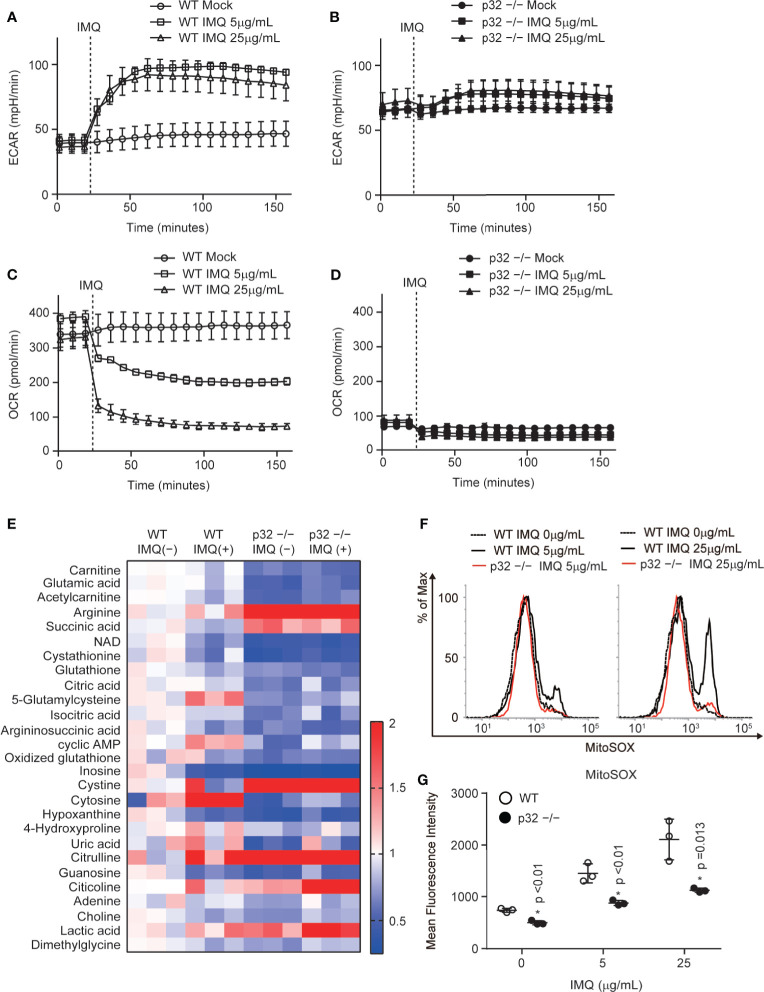
p32/C1qbp regulates mitochondrial metabolism and ROS after IMQ stimulation. **(A–D)** Real-time changes in the ECAR **(A, B)** or OCR **(C, D)** in WT **(A, C)** and p32^−/−^ **(B, D)** DCs treated with 5 μg/mL IMQ. Vertical dotted lines indicate the initiation of treatment. Data are shown as means ± SDs of triplicate wells. **(E)** Comparisons of the amounts of metabolites between WT and p32^−/−^ BMDCs with or without IMQ stimulation. Heat map of WT and p32^−/−^ DC metabolites that exhibited statistically significant changes (P < 0.05). **(F, G)** Flow cytometry histograms **(F)** and quantification **(G)** of the expression of mitochondrial ROS (MitoSOX) in WT and p32^−/−^ DCs treated with 5 or 25 μg/mL IMQ. Data are shown as means ± SDs of triplicate wells. Data are representative of two **(E)** and three **(A–D, F–G)** independent experiments.

Because we found that p32/C1qbp is involved in IMQ-induced DC activation *in vitro* (**Figure 3**), we investigated the underlying molecular and metabolic mechanisms. IMQ stimulation induces glycolytic and mitochondrial metabolic changes in DCs. Thus, we examined whether IMQ influenced glycolysis and mitochondrial oxygen consumption using a flux analyzer assay. We measured the extracellular acidification rate (ECAR) as an index of lactate production and glycolysis in BMDCs; we also measured the oxygen consumption rate (OCR) as an indicator of mitochondrial respiration in BMDCs. Indeed, we found that the ECAR rapidly increased in WT DCs that had been subjected to IMQ stimulation (**Figure 4A**). In contrast, the change in a glycolytic flux by IMQ stimulation was negligible in p32^–/–^ DCs (**Figure 4B**). We also found that the OCR rapidly decreased in WT DCs that had been subjected to IMQ stimulation (**Figure 4C**). In contrast, the OCR in p32^–/–^ DCs was severely suppressed due to the inhibition of mitochondrial translation and resultant mitochondrial respiratory chain complex I deficiency, and therefore was not changed by IMQ injection (**Figure 4D**). While the mitochondrial mass of WT DCs was increased by IMQ, the mass in p32^–/–^ DCs originally increased and was little affected by IMQ (**Supplementary Figure 4A**). The mitochondrial membrane potential was similarly decreased after IMQ stimulation both in WT and p32^–/–^ DCs. (**Supplementary Figure 4B**). To explore the detailed metabolic mechanism by which p32 controls IMQ-dependent DC activation, we compared intracellular metabolites between WT and p32^–/–^ DCs. Indeed, metabolites of the fatty acid oxidation (carnitine and acetylcarnitine) and tricarboxylic acid (TCA) cycle (citric acid and isocitric acid) were significantly reduced in p32^–/–^ DCs before IMQ stimulation (**Figure 4E** and **Supplementary Figures 4C, D**). Furthermore, the metabolites of glutathione metabolism (glutamic acid, glutathione, 5-glutamylcysteine, and oxidized glutathione) were significantly lower in p32^–/–^ DCs (**Figure 4E**). Because the concentration of oxidized glutathione was decreased in p32^–/–^ DCs, we expected that the generation of mtROS would be decreased in p32^–/–^ DCs.

IMQ-induced inflammasome activation requires mtROS through mitochondrial respiratory chain complex I activity. In addition, IMQ induces the production of IL-1β through mtROS- and caspase-1-dependent NLRP3 inflammasome activation. Indeed, we found that the inhibition of mitochondrial respiratory chain complex I could suppress a burst of mitochondrial ROS by IMQ stimulation, as well as the production of IL-1β and IL-23 in WT DCs (**Supplementary Figures 5A–C**). Because IMQ stimulation induces a burst of mitochondrial ROS, we compared IMQ-induced mtROS between WT and p32^–/–^ DCs. We found that the level of IMQ-induced mtROS was decreased in p32^–/–^ DCs (**Figures 4F, G**). In addition, IMQ-induced caspase-1 activation was decreased in p32^–/–^ DCs (**Figure 3D**). These findings indicate that p32/C1qbp supports IMQ-induced IL-1β through mtROS *in vitro*.

### Mitochondrial ROS Promote IMQ-Induced DC Activation *In Vitro*

**Figure 5:**
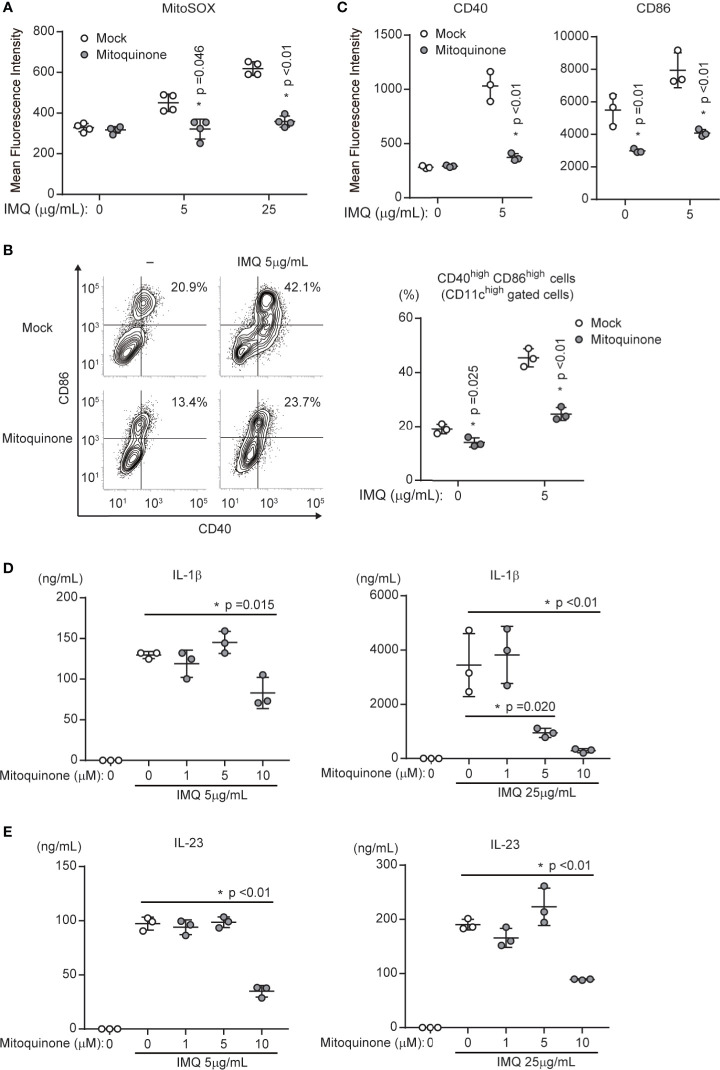
Mitochondrial ROS are required for DC activation by IMQ stimulation. **(A)** WT DCs in the presence or absence of mitoquinone were stimulated with 5 or 25 μg/mL IMQ and analyzed for the expression of mitochondrial ROS (MitoSOX). Data are shown as means ± SDs (n = 4). **(B, C)** WT DCs in the presence or absence of mitoquinone were stimulated with IMQ and analyzed to quantify the expression of cell surface markers CD86 and CD40 **(C)**. Numbers in the top right corners indicate the percentages of CD86^+^ CD40^+^ cells (**B**, left). Pooled results from three independent experiments are shown (**B**, right). Data are shown as means ± SDs of triplicate samples. **(D, E)** Cytokine production was compared among WT DCs exposed to mock treatment or mitoquinone. Cells were stimulated with 5 or 25 μg/mL IMQ for 24 h in the presence or absence of inhibitors. Data indicate the levels of IL-1β **(D)** and IL-23 **(E)** in cell culture supernatants (means ± SDs of triplicate wells). Data are shown as means ± SDs. *p < 0.05 versus mock treatment. Data are representative of three independent experiments.

We showed that p32/C1qbp supports IMQ-induced DC activation (**Figure 3**). In addition, the loss of p32/C1qbp suppressed the generation of mtROS after IMQ stimulation (**Figure 4**). Thus, we presumed that mtROS could influence DC activation by IMQ stimulation. Indeed, we found that MitoQ, a mitochondria-targeted antioxidant that reduces mtROS, could suppress IMQ-induced mtROS (**Figure 5A**). In addition, MitoQ inhibited IMQ-induced surface expression of both CD40 and CD86 in WT DCs (**Figures 5B, C**). Although five μM MitoQ was not sufficient to suppress the production of IL-1β and IL-23, treatment with ten μM MitoQ significantly suppressed the production of IL-1β and IL-23 after IMQ stimulation (**Figures 5D, E**). These results indicate that the inhibition of mtROS suppresses IMQ-induced DC activation *in vitro*.

### Inhibition of Mitochondrial ROS Suppresses IMQ-Induced Psoriatic Inflammation

**Figure 6:**
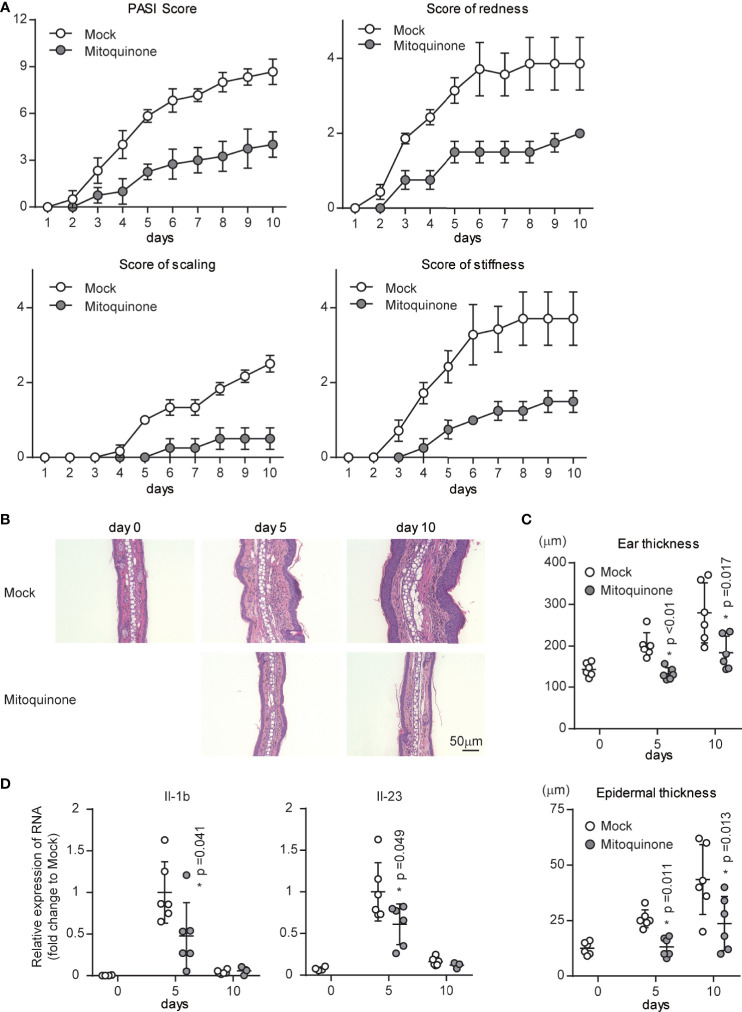
Effects of a mitochondrial ROS inhibitor on psoriatic inflammation *in vivo*. WT mice were topically treated with IMQ for 9 consecutive days and received either mitoquinone or mock treatment every 2-3 days by intraperitoneal injection, beginning on day 0. **(A)** Cumulative Psoriasis Area Severity Index (PASI) scores were calculated based on the individual scores for redness, scaling, and stiffness daily until 9 days after IMQ application. Data are shown as means ± SDs (n = 4–6). **(B, C)** Microscopy of cross-sections of mouse ears stained with hematoxylin and eosin **(B)**. Changes in ear (**C** upper) and epidermal (**C** lower) thickness over time. Scale bars, 50 µm. **(D)** Real-time PCR analysis was performed to assess mRNA levels in IMQ-treated mouse ears on days 5 and 10 (n ≥ 3). The results were normalized to 18S expression and are shown as means ± SDs. *p < 0.05 versus mock treatment. Data are representative of three independent experiments.

Our data indicated that p32/C1qbp supports IMQ-induced DC activation through mtROS *in vitro* (**Figures 4** and **5**). To examine the role of mtROS in IMQ-induced psoriatic inflammation, we analyzed psoriatic symptoms in ears that had been subjected to repetitive topical application of IMQ after intraperitoneal injection of MitoQ (10 mg/kg) in WT mice. We found that psoriatic symptom severity was reduced in mice treated with MitoQ, compared with WT mice that had received mock treatment (**Figure 6A**). Histological examination also revealed that IMQ-treated skin from WT mice with MitoQ exhibited reduced epidermal hyperplasia with massive infiltration of inflammatory cells (**Figures 6B, C**). Consistent with our *in vitro* data (**Figures 5D, E**), the expression levels of Il-1b and Il-23 in the ears of WT mice treated with MitoQ were significantly decreased on day 5 after IMQ treatment (**Figure 6D**). Although we could not determine whether MitoQ indeed suppressed mtROS in DCs, the inhibition of mtROS was sufficient to suppress IMQ-induced psoriatic inflammation.

## Discussion

p32/C1qbp is a gene associated with psoriasis in humans (**Figure 1A**). Our mouse genetics study revealed the contribution of a p32/C1qbp-dependent mechanism to the exacerbation of psoriasis. We also showed that mtROS-dependent IL-1β is involved in the exacerbation of psoriatic inflammation. The inhibition of mtROS was effective for reducing disease severity in our mouse model of psoriasis (**Figure 6**). Our findings suggest that p32/C1qbp, which functions as a multifunctional chaperone protein in mitochondria, is an important genetic factor involved in psoriasis.

Previous studies showed that DC activation by TLR agonists contributes to the exacerbation of several diseases (e.g., autoimmune diseases, inflammatory diseases, allergies, and cancer) (20, 45–47). In particular, DCs activated by TLR agonists reportedly induce Th17 differentiation and exacerbate autoimmune diseases (e.g., psoriasis and experimental autoimmune encephalomyelitis) (48, 49). The mechanism by which IMQ-treated DCs induce Th17 differentiation in psoriatic inflammation has been clarified by recent studies (22, 30, 50). Although our study does not fully show the mechanism underlying the exacerbation of psoriasis through IMQ-induced DC activation, we have elucidated a portion of the mechanism underlying exacerbation of psoriasis by p32/C1qbp and mtROS-dependent pathways. To clarify the relationship between p32/C1qbp and psoriatic inflammation, it will be necessary to cross CD11c-cre and p32 flox mice and analyze them using a psoriatic model.

Previous studies showed that IL-1β is involved in the pathogenesis of psoriasis and psoriatic arthritis (5, 39, 51, 52). Although the inhibition of IL-1β is not fully established as an effective treatment for psoriasis, treatment with IL-1β inhibitors reportedly improved disease in several patients with psoriasis (53, 54). Because IL-1β has a critical role in the differentiation and activation of IL-17-producing T cells (55–57), IL-1β may be directly or indirectly involved in the exacerbation of psoriasis by promoting Th17 cell polarization. A previous study showed that DCs in IMQ-treated skin mainly produce IL-1β (52); the study also showed that the IL-1β–IL-1R signaling pathway contributes to skin inflammation and psoriasis pathogenesis. Despite differences in major IL-1β-producing cells between mouse models and human disease, we speculate that the inhibition of IL-1β production can be a treatment for psoriasis, similar to the inhibition of IL-17 production.

ROS are reportedly pathogenic in psoriasis (58–60). In addition, IMQ-induced psoriatic dermatitis can be prevented by ROS (61). Although there are many sources of ROS (e.g., NADPH oxidases [NOX], mitochondrial ETC, xanthine oxidase, uncoupled endothelial nitric oxide synthase [eNOS], cytochrome P-450 oxygenase, and cyclooxygenase), there remains a lack of clarity regarding the specific ROS pathway involved in the exacerbation of psoriasis (60, 62). In this study, we determined that mtROS suppression attenuates the exacerbation of psoriasis in a mouse model (**Figure 6**). The generation of mtROS by IMQ stimulation may promote the production of inflammatory cytokines, as well as DC maturation. Because MitoQ, an inhibitor of mtROS, also suppressed IMQ-induced DC activation *in vitro* (**Figure 5**), mtROS inhibition may serve as a therapeutic target for immune disorders associated with DC activation. Importantly, we could not analyze the roles of keratinocytes in psoriasis pathogenesis. A previous study demonstrated the critical role of mtROS from the mitochondrial ETC during keratinocyte differentiation (63). However, the role of mtROS in psoriasis-associated keratinocytes is unknown. Elucidation of the role of mtROS in keratinocytes may be important for the development of psoriasis treatments. Furthermore, several studies have shown that mtROS promote Th17 cell generation and exacerbation of Th17-related autoimmune disease (64, 65). Based on the prior results and our findings in this study, mtROS may be involved in the activation of DCs and Th17 cells in autoimmune diseases. Therefore, we anticipate that the inhibition of mtROS will be a useful therapeutic target for DC- and Th17-related autoimmune diseases.

The relationship between mitochondria and psoriasis has been unclear thus far. In this study, we have demonstrated that p32/C1qbp is an important factor for the exacerbation of psoriasis through mtROS. Because p32/C1qbp is a multifunctional chaperone protein in mitochondria (66), we suspect that p32/C1qbp is involved in other psoriasis-related pathways. Several previous studies showed that p32/C1qbp regulates mitochondrial translation and metabolism (34, 35, 67). Because metabolites of fatty acid oxidation and the TCA cycle were decreased in p32-deficient DCs (**Figure 4E**), intracellular metabolism involving fatty acid oxidation and the TCA cycle may contribute to the exacerbation of psoriasis. A recent study demonstrated that intracellular metabolism of fatty acids and mtROS induced IL-23-mediated psoriatic inflammation through DC activation (30). The present study also showed that mtROS induce IL-1β and IL-23-mediated psoriatic inflammation. However, our study did not show a relationship between mitochondrial metabolism and psoriasis. Therefore, further research is required regarding the relationship between autoimmune diseases and mitochondrial metabolism.

In summary, we have demonstrated that the mitochondrial protein p32/C1qbp has important roles in the exacerbation of psoriasis and DC activation. Recent studies in mice revealed that p32/C1qbp is involved in erythropoiesis, innate immunity, and the integrated stress response (34, 41, 68). Moreover, p32/C1qbp transcription patterns are associated with the outcomes of influenza A, actinic keratosis, and cancers (69–71). Considering the varied expression of p32/C1qpb in many cell types and organs, we promote further study on the association between p32/C1qbp and mtROS focusing on the pathogenesis of autoimmune diseases in mice and humans. In particular, because p32/C1qbp and mtROS may have important roles in autoimmune diseases, further analyses are essential.

## Limitations of the Study

There were a few limitations in this study. First, we could not fully rule out the indirect effects of epithelial cells in the mouse model. Second, we could not measure the expression of p32/C1qbp in a group of patients with psoriasis, although past microarray data suggest that p32/C1qbp is involved in the exacerbation of psoriasis (38). Third, we could not fully demonstrate mechanistic links between the p32/C1qbp-mtROS-inflammasome and DC maturation. Therefore, further investigations are required to conclusively determine the functions of p32/C1qbp in psoriasis.

## Data Availability Statement

The datasets presented in this study can be found in online repositories. The names of the repository/repositories and accession number(s) can be found in the article/**Supplementary Material**.

## Ethics Statement

The animal research protocols were approved by the Committee of Ethics on Animal Experiments, Faculty of Medical Sciences, Kyushu University.

## Author Contributions

KG and DK designed the study. SM, KG, YN, DS, and YT carried out the experiments. SM and KG prepared the figures and wrote the manuscript. SO and DK supervised the experiments. KG, SO, and DK reviewed the experiments. All authors contributed to the article and approved the submitted version.

## Funding

This work was supported by JSPS KAKENHI Grant Numbers JP18K11077 and JP16K19196 (both to KG) and JP20H00530 and JP17H01550 (both to DK). This work was also supported by grants from the Takeda Science Foundation, The Shin-Nihon Foundation of Advanced Medical Research, and Charitable Trust Laboratory Medicine Research Foundation of Japan (to KG).

## Conflict of Interest

The authors declare that the research was conducted in the absence of any commercial or financial relationships that could be construed as a potential conflict of interest.

## Publisher’s Note

All claims expressed in this article are solely those of the authors and do not necessarily represent those of their affiliated organizations, or those of the publisher, the editors and the reviewers. Any product that may be evaluated in this article, or claim that may be made by its manufacturer, is not guaranteed or endorsed by the publisher.
