# Supplementary material for: Mitochondrial Reactive Oxygen Species Are Essential for the Development of Psoriatic Inflammation
Source: Front Immunol. 2021 Aug 6;12:714897. doi: 10.3389/fimmu.2021.714897 (PMC8378889; doi:10.3389/fimmu.2021.714897)
Supplement: Supplementary file 1 [file DataSheet_1.docx]

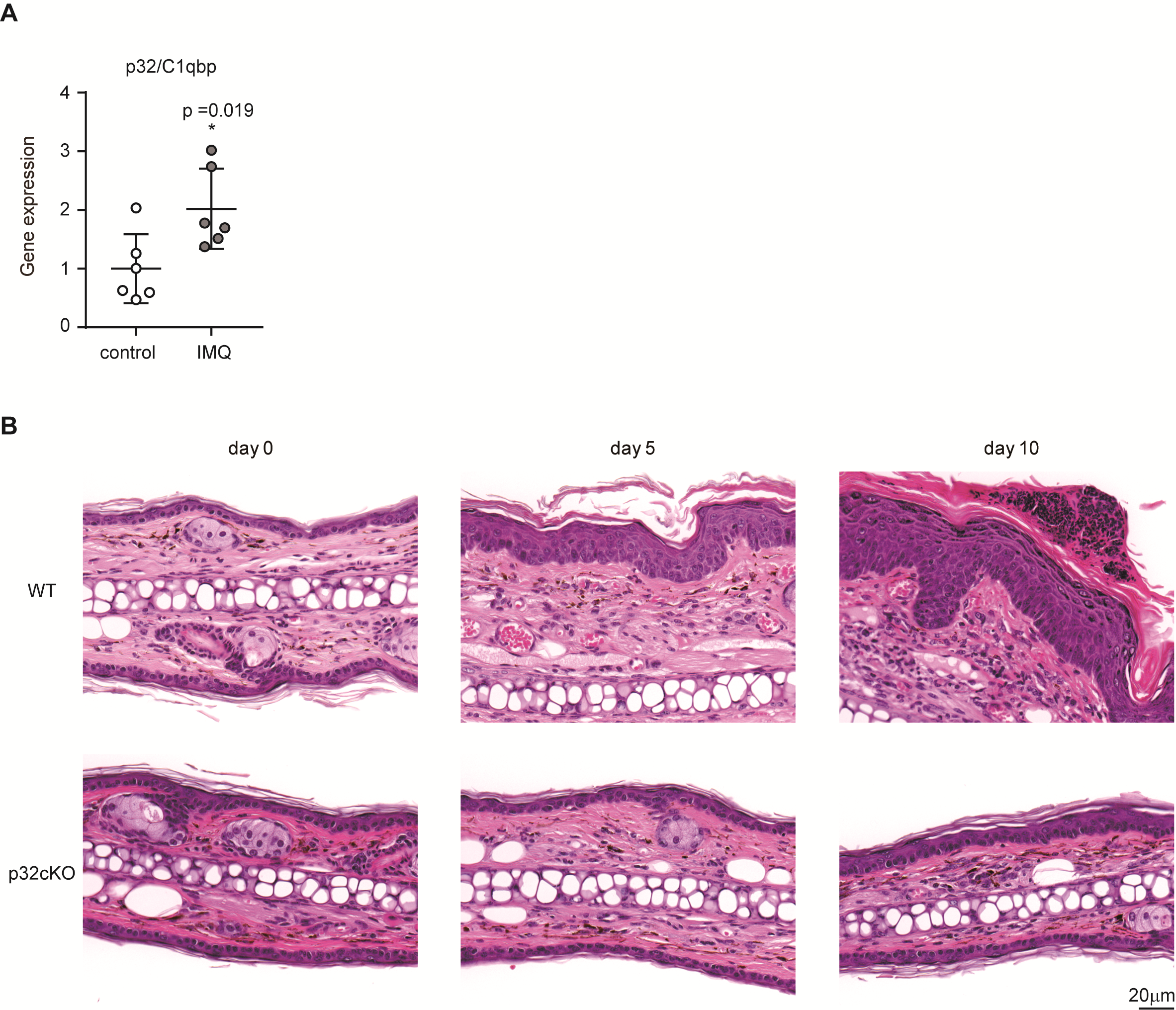


**Supplementary Figure 1. Expression levels of p32/C1qbp**
(A) WT mice were topically treated with IMQ-containing cream for 4 consecutive days, and the expression levels of p32/C1qbp in ears were analyzed by real-time PCR (n = 6 mice per group). The results were normalized to 18S expression and are shown as means ± SDs. (B) Microscope images of cross-sections of mouse ears stained with hematoxylin and eosin. Scale bars, 20 µm. Data are representative of three (A, B) independent experiments.


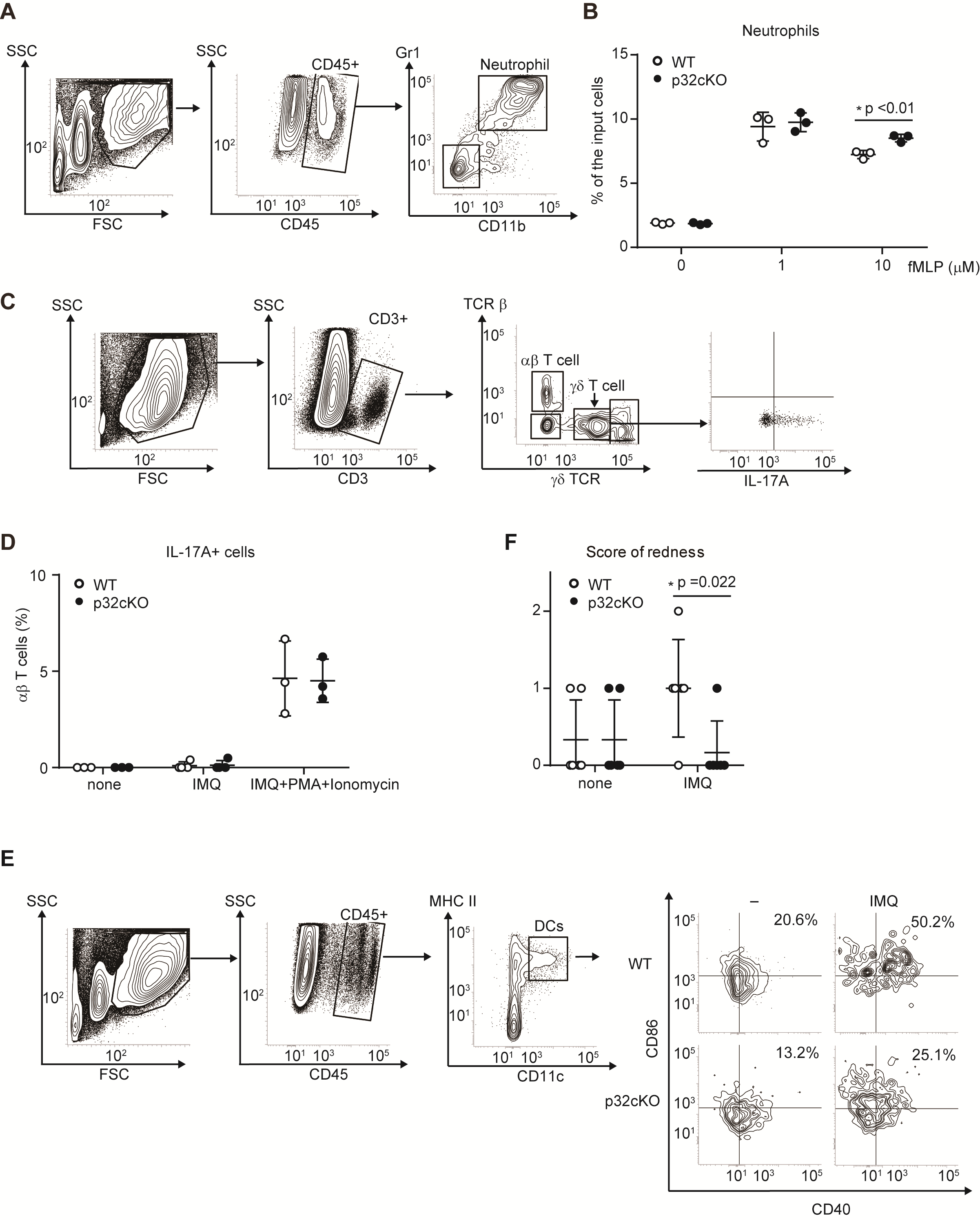


**Supplementary Figure 2. Gating strategies for FACS analysis**
(A, C, E) Representative gating strategies for neutrophils (A), γδ T cells (C), and DCs (E). (B) Chemotactic responses of BM neutrophils to fMLP were compared between WT and p32cKO mice in a Transwell chemotaxis assay. The results are expressed as the percentage of the input cells (mean ± SD of triplicate wells). (D) WT and p32cKO mice (n ≥ 3) were treated with IMQ for 4 consecutive days. IL-17^+^ αβ T cell numbers were analyzed before (E, left and middle lanes) and after (E, right lane) PMA/ionomycin (PI) stimulation *in vitro*. Results are expressed as means ± SDs. (F) IMQ-untreated or treated BMDCs from WT or p32cKO mice were subcutaneously injected into WT mice (n=6). Ear redness was analyzed at 48 h after injection. Results are expressed as means ± SDs. Data are representative of two (B, F) and three (A, C–E) independent experiments.


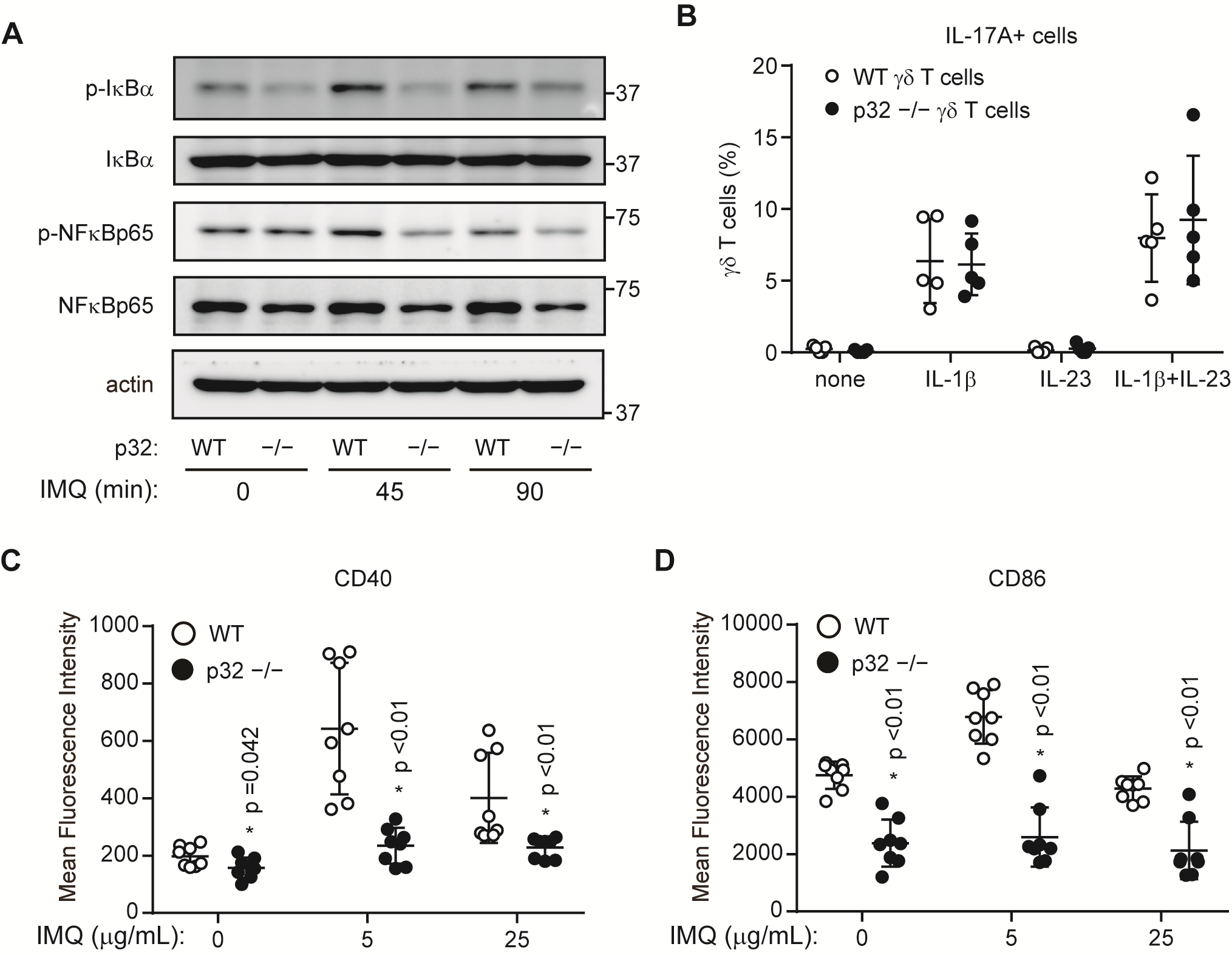


**Supplementary Figure 3. Expression levels of CD40 and CD86 in BMDCs**
(A) WT and p32^−/−^ DCs were stimulated with 5 μg/mL IMQ for the indicated intervals and analyzed to quantify the phosphorylation of IκBα and NFκBp65. β-Actin was used as an internal control. (B) IL-17^+^ γδ T cell populations in the ears were analyzed by flow cytometry. IL-17^+^ γδ T cell numbers were analyzed before (left lane) and after (right lane) IL-1β, IL-23, and IL1β+IL-23 stimulation *in vitro*. (C, D) Expression levels of CD40 (C) and CD86 (D) cell surface markers in WT (upper) and p32^−/−^ DCs stimulated for 12 h with IMQ and analyzed using flow cytometry. Data are shown as means ± SDs. *p < 0.05 versus WT. Data are representative of two (A, B) and three (C, D) independent experiments.


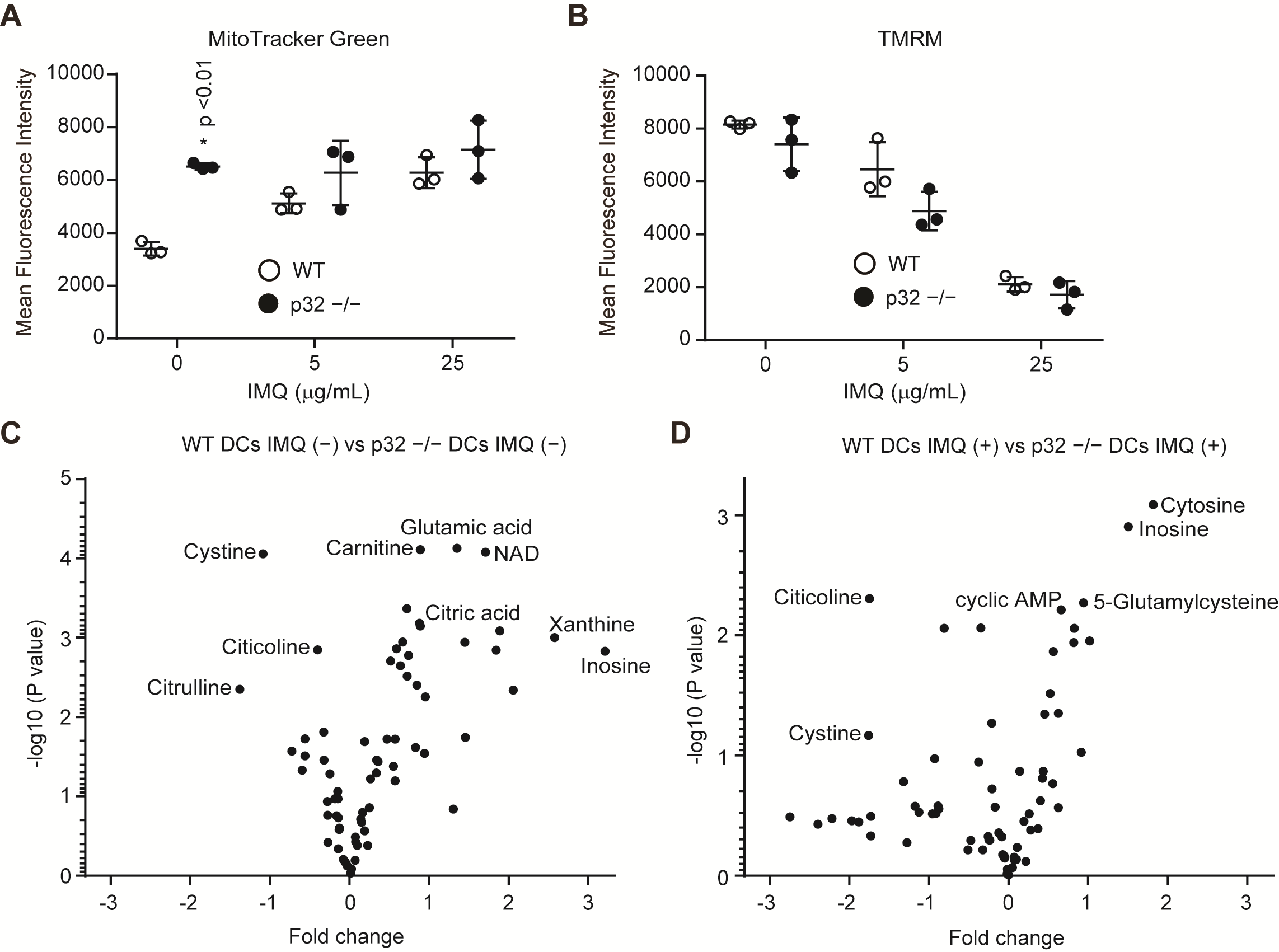


**Supplementary Figure 4. Comparisons of metabolite quantities between WT and p32^−/−^ DCs**
(A, B) Mitochondrial masses were measured using MitoTracker Green FM (A) and mitochondrial membrane potentials (MMP) were measured using TMRM (B) in BMDCs treated with IMQ stimulation, by means of FACS analysis. Data are shown as means ± SDs of triplicate samples. *p < 0.05, versus WT DCs. (C, D) Comparisons of the amounts of metabolites between WT and p32^−/−^ DCs without (C) and with (D) IMQ stimulation. Volcano plot showing differential amounts between WT (n = 3) and p32^−/−^ DCs (n = 3). Fold change is calculated as log2 (expression in WT DCs / expression in p32^−/−^ DCs). Data are shown as means ± SDs (A, B). *p < 0.05 versus WT. Data are representative of two (A-D) independent experiments.


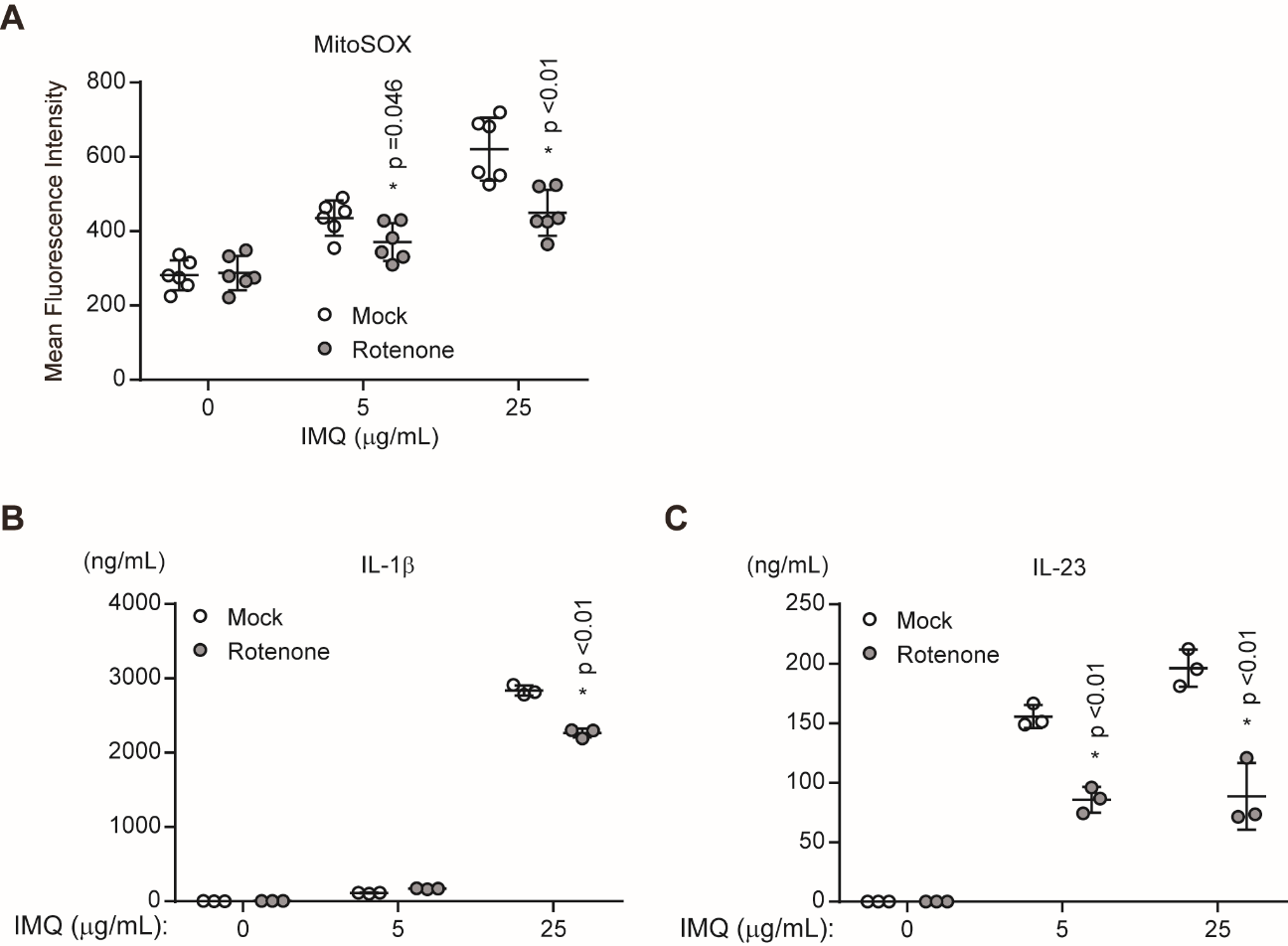


**Supplementary Figure 5. Rotenone suppresses IMQ-induced IL-1β production in BMDCs**
(A) WT DCs in the presence or absence of rotenone (2.5 μM) were stimulated with 5 or 25 μg/mL IMQ and analyzed to quantify the expression of mitochondrial ROS (MitoSOX). Data are shown as means ± SDs of triplicate samples. (B, C) Cytokine production was compared among WT DCs exposed to mock treatment or rotenone. Cells were stimulated with 5 or 25 μg/mL IMQ for 24 h in the presence or absence of inhibitors. Data indicate the levels of IL-1β and IL-23 in cell culture supernatants (means ± SDs of triplicate wells). Data are shown as means ± SDs. *p < 0.05 versus mock treatment. Data are representative of two (A-C) independent experiments.

Supplemental Table S1 List of primer sequences used for RT-PCR analysis

| Primer name | Sequence 5’→3’ |
| --- | --- |
| 18S Forward | CGCGGTTCTATTTTGTTGGT |
| 18S Reverse | AGTCGGCATCGTTTATGGTC |
| S100a8 Forward | AAATCACCATGCCCTCTACAAG |
| S100a8 Reverse | CCCACTTTTATCACCATCGCAA |
| Il-17a Forward | AGCTGGACCACCACATGAAT |
| Il-17a Reverse | AGCATCTTCTCGACCCTGAA |
| Il-17f Forward | ATGAAGTGCACCCGTGAAACAG |
| Il-17f Reverse | CTCAGAATGGCAAGTCCCAACA |
| Il-17c Forward | CTCCTGCTTCTAGGCTGGTTG |
| Il-17c Reverse | CCACCTGGCACTTCGAGTTAG |
| Il-22 Forward | CCGAGGAGTCAGTGCTAAGG |
| Il-22 Reverse | GTAGGGCTGGAACCTGTCTG |
| Il-23 Forward | CCAGCGGGACATATGAATCT |
| Il-23 Reverse | TGTGGGTCACAACCATCTTC |
| Il-6 Forward | TGATGCACTTGCAGAAAACA |
| Il-6 Reverse | ACCAGAGGAAATTTTCAATAGGC |
| Il-1b Forward | GCTTCAGGCAGGCAGTATCAC |
| Il-1b Reverse | CGACAGCACGAGGCTTTTT |
| Il-12b Forward | ACATCTACCGAAGTCCAATGCA |
| Il-12b Reverse | GGAATTGTAATAGCGATCCTGAGC |
| Ifn-b1 Forward | CTGGCTTCCATCATGAACAA |
| Ifn-b1 Reverse | CATTTCCGAATGTTCGTCCT |

Supplemental Table S2 List of antibodies used for flow-cytometry

| REAGENT | SOURCE | IDENTIFIER | DILUTION |
| --- | --- | --- | --- |
| anti-CD45 antibody | Tonbo Biosciences | Cat# 75-0451, RRID:AB_2621947 | 1:100 |
| anti-Gr-1 antibody | BioLegend | Cat# 108416, RRID:AB_313381 | 1:1000 |
| anti-CD11b antibody | BioLegend | Cat# 101226, RRID:AB_830642 | 1:500 |
| anti-TCR γδ  antibody | BioLegend | Cat# 118108, RRID:AB_313832 | 1:200 |
| anti-TCR β  antibody | BioLegend | Cat# 109228, RRID:AB_1575173 | 1:200 |
| anti-IL-17A  antibody | BioLegend | Cat# 506908, RRID:AB_536010 | 1:50 |
| Anti-CD3 antibody | BioLegend | Cat# 100236, RRID:AB_2561456 | 1:200 |
| anti-CD11c antibody | BioLegend | Cat# 117310, RRID:AB_313779 | 1:200 |
| anti-CD40 antibody | BioLegend | Cat# 124608, RRID:AB_1134096 | 1:100 |
| anti-CD40 antibody | BioLegend | Cat# 124608, RRID:AB_1134096 | 1:400 |

Supplemental Table S3 List of antibodies used for western blot

| REAGENT | SOURCE | IDENTIFIER | MW(kD) | DILUTION |
| --- | --- | --- | --- | --- |
| anti-IL-1β antibody | Cell Signaling Technology | Cat# 31202, RRID:AB_2799001 | 17, 31 | 1:2000 |
| anti-Cleaved-Caspase-1 antibody | Cell Signaling Technology | Cat# 89332 | 22 | 1:2000 |
| anti-Caspase-1 antibody | Cell Signaling Technology | Cat# 24232, RRID:AB_2890194 | 48 | 1:2000 |
| anti-β-actin antibody | Merck (Sigma-Aldrich) | Cat# A5441, RRID:AB_476744 | 42 | 1:5000 |
| anti-p-IKKα/β  antibody | Cell Signaling Technology | Cat# 2697, RRID:AB_2079382 | 85, 87 | 1:2000 |
| anti-IKKα  antibody | Cell Signaling Technology | Cat# 2682, RRID:AB_331626 | 85 | 1:2000 |
| anti-IKKβ  antibody | Cell Signaling Technology | Cat# 2678, RRID:AB_2122301 | 87 | 1:2000 |
| anti-p32 antibody | Dongchon Kang, Kyushu University |  | 32 | 1:2000 |
| anti-p-IκBα antibody | Cell Signaling Technology | Cat# 2859, RRID:AB_561111 | 39 | 1:2000 |
| anti-IκBα antibody | Cell Signaling Technology | Cat# 4814, RRID:AB_390781 | 39 | 1:2000 |
| anti-p-NF-κB antibody | Cell Signaling Technology | Cat# 3033, RRID:AB_331284 | 65 | 1:2000 |
| anti-NF-κB antibody | Cell Signaling Technology | Cat# 8242, RRID:AB_10859369 | 65 | 1:2000 |
